# How do factors of sociodemographic, health literacy and dementia experience influence carers’ knowledge of dementia?

**DOI:** 10.1177/14713012221074219

**Published:** 2022-03-02

**Authors:** Sophie Crawley, Kirsten Moore, Victoria Vickerstaff, Emily Fisher, Claudia Cooper, Elizabeth L Sampson

**Affiliations:** Marie Curie Palliative Care Research Department, Division of Psychiatry, 325312 UCL, London, UK; Division of Psychiatry, 4919 UCL, London, UK; Barnet, Enfield and Haringey Mental Health Trust, London, UK

**Keywords:** carers, family, dementia, knowledge, health literacy

## Abstract

**Background:**

Dementia is a life limiting disease following a progressive trajectory. As carers often become key decision makers, their knowledge of dementia will have health implications for the person living with dementia as well as carer’s psychological wellbeing.

**Aim:**

To explore how sociodemographic factors, health literacy and dementia experience influence family carers knowledge about dementia.

**Method:**

In this cross-sectional, mixed methods study, we interviewed 150 family carers and assessed their dementia knowledge using the Dementia Knowledge Assessment Scale (DKAS). Linear regression analyses were used to examine whether health literacy, previous experiences of dementia, support group attendance and sociodemographic characteristics predicted knowledge. Sixteen carers also completed qualitative interviews which explored unmet information needs. Transcripts and field notes were thematically analysed.

**Results:**

Most participants were partners (47%) or adult children (48%) and cared for someone with severe (32%) or moderate (43%) dementia. Mean DKAS scores were 34.8/50 (SD = 7.0, range = 17–48) reflecting 8/25 incorrect answers. Backwards elimination regression found greater dementia knowledge was associated with greater health literacy for appraising information (coef 3.48, 95% CI (1.38, 5.58); *p* = 0.001) and more years of education (coef 0.39, 95% CI (0.12, 0.65); *p* = 0.004). Although not significant, knowledge was slightly lower in those who attended a support group, and a trend was found between ability to understand health information and knowledge. Only 39% accurately identified dementia as life shortening, indicating notable gaps in knowledge. Four qualitative themes were identified; arm yourself with information, ability to steer through information, other experience of dementia can be helpful and the importance of relationships with health care professionals.

**Conclusions:**

In an information age, vast amounts of information are available, but this can bring difficulties. Carers with more years of education and higher health literacy knew more about dementia. Professionals should consider how carers with lower health literacy can be supported through provision of timely, relevant information.

## Introduction

Unpaid carers, usually family members or friends referred to as carers from hereon, provide nearly half the total cost of dementia care in the United Kingdom (Prince et al., 2014) and are identified as ‘the most valuable resource for people with dementia’ (Health, 2009). The economic impact of dementia and how people with dementia and their families will be supported as prevalence increases has become a public health priority both globally and in the United Kingdom (World Health Organization, 2012).

Due to the progressive cognitive decline in dementia, carers are often involved in decision making and care planning for the person living with dementia (High & Rowles, 1995). This ‘progressive surrogacy’ highlights the necessity of carers having knowledge of dementia, including scientific knowledge and understanding of the disease cause, consequences, evolution and treatment (Cahill et al., 2015). Greater knowledge of dementia is also associated with fewer unnecessary interventions, is predictive of higher levels of comfort at end of life for the person living with dementia and serves as a foundation for care planning (Mitchell et al., 2009; Robinson et al., 2014; van der Steen et al., 2013). Additionally, knowledge can have implications for carer psychological wellbeing as feeling unprepared for the death of the person living with dementia has been associated with prolonged grief, depression and anxiety (Hebert et al., 2006). Key elements of feeling prepared have been identified as understanding the prognosis and recognising of symptoms of decline. Therefore, greater knowledge should increase preparation, which in turn may benefit carers’ mental health (Moore et al., 2019).

A number of socio-economic factors have been identified as influencing carers’ knowledge of dementia. Health literacy defined as ‘…the cognitive and social skills which determine the motivation and ability of individuals to gain access to, understand and use information in ways which promote and maintain good health’ (Osborne et al., 2013) is likely to play a pivotal role in carers’ knowledge. Low health literacy has been associated with difficulties managing health, less effective communication with health and social care practitioners (Roberts, 2015) and poor disease knowledge in long-term health conditions (Williams et al., 1998). Forty-three percent of working age UK adults are unable to make use of everyday health information (Rowlands et al., 2015), indicating that a substantial number of individuals may not understand health information related to dementia and the person they are caring for. A further identified risk factor for poor literacy and poor health literacy is deprivation (Saranjit & Lennard, 2004) which may also influence dementia knowledge. Whilst older age has also been associated with lower dementia knowledge in dementia family carers (Cahill et al., 2015; Werner, 2001), a systematic review of the public’s knowledge of dementia found that only three of the 15 included studies found an inverse relationship between dementia knowledge and age (Cahill et al., 2015). Lower education has also been associated with lower dementia knowledge (Cahill et al., 2015; Werner, 2001).

It is less clear whether dementia severity or duration of care influences dementia knowledge. Some evidence indicates that length of time caring is not correlated with dementia knowledge (Werner, 2001). Also, dementia is not recognised as a terminal condition or potential cause of death across all carers, regardless of duration of stay on a Dementia Specific Unit or level of cognitive decline (Andrews et al., 2017). However, Carpenter et al. found that other experience of dementia, either in a personal, professional or voluntary capacity, was associated with greater knowledge (Carpenter et al., 2011), with past experience being described as the ‘foundation of knowledge’ (Carter et al., 2018).

In the United Kingdom, dementia support groups are widely available and offer the opportunity for information and knowledge sharing between carers and from facilitator to attendees. Attendance at a class, educational programme or support group has been significantly associated with more knowledge (Carpenter et al., 2011), and qualitative findings endorse that support groups provided information and advice to carers, as well as discussion of needs and helped prepare them to face the person living with dementia’s further deterioration (Shanley et al., 2013). However, a more recent study reflects that whilst carers may benefit from practical information and support from carer support groups, the type of information they access and whether this supports their understanding of the dementia trajectory has not been explored (Andrews et al., 2017).

Clinical guidelines and national strategies reflect the importance of carers having knowledge of the condition and what to expect and recommend that education on the progressive and terminal nature of dementia is provided (van der Steen et al., 2014). Many studies have examined education interventions for carers (*removed for review (Moore, 2019)) but few have examined what factors are most strongly associated with dementia knowledge.

We aimed to determine how factors of sociodemographic, health literacy and dementia experience predict dementia knowledge in family carers to inform future carer educational interventions. We selected factors we anticipated would influence dementia knowledge based on the previous research cited above. Our secondary aim was to explore carers’ experiences of how knowledge was acquired and factors they consider contributing to their knowledge.

## Method

This study utilised data collected through a wider programme of work exploring preparation for end of life and pre-death grief in carers of people living with dementia; study name removed (main findings reported separately, *ref removed for review (Moore, 2019). Briefly, study name removed was a cross-sectional mixed methods study of family carers of people with dementia who were currently providing care or support (practical, social, emotional or supervisory) for a person living with any diagnosed dementia. More than one carer in a family were able to participate. Carers had to be aged 18 and over, living in England or Wales and could be caring for a person with dementia living at home or in a care home. Carers who were not able to communicate in English or did not have the capacity to provide written informed consent were excluded. Carers were assessed for eligibility by telephone or email.

### Ethics

Ethics approval was obtained through the *removed for review London - South East Research Ethics Committee (Reference 17/LO/1881) and *removed for review University College London Research Ethics Committee (Reference 11,755/001). The study was also approved by the Health Research Authority (Sponsor Reference Number 17/0477).

### Recruitment

Carers were recruited via several avenues including; 9 NHS hospital trusts across England, the Join Dementia Research (JDR) Web site and through Alzheimer’s Society including their research network and Care and Cure magazine. All carers were provided with a Participant Information Sheet (PIS) and were given 3 days (or as required) to read this before being contacted via phone or email to schedule the interview if they agreed to participate. Participants invited to take part in an additional semi-structured interview were purposively selected to include variation in gender, relationship type and dementia severity.

### Procedure

Participants completed the Dementia Knowledge Assessment Scale (DKAS) alongside a range of standardised questionnaires during interviews that were conducted face-to-face at the participant’s home, at UCL or other quiet location of the participant’s choice. The interview included additional open-ended/semi-structured questions which explored dementia knowledge, avenues of support and preparation for the end of life for their friend or relative with dementia. All responses were recorded on a paper Case Report Form during the interview.

The carer provided written consent immediately before the interview. The first interview involving the completion of standardised measures was not audio-recorded as open-ended responses were anticipated to be minimal. However, once data collection commenced, we found participants were providing relevant contextual and additional information; therefore, field notes were taken during the interview and developed into a summary following the interview. All interviews were conducted by * and * SC and KM *removed for review* between January 2018 and January 2019 and ranged between 1 and 3 h.

A subsample of participants were invited to take part in an additional semi-structured interview which was audio recorded and explored the topics in more detail. Participants gave permission for the interviews to be recorded using an encrypted digital recorder; audio recordings were transcribed verbatim. Data relevant to the research aims from both the first and second interview are reported here.

### Measures

Dementia knowledge: the DKAS was completed to provide a total score of dementia knowledge, and measured knowledge across four domains; Causes and characteristics, Communication and behaviours, Care considerations and Risks and health promotion. The DKAS has been subjected to a confirmatory factory analysis which demonstrated the reliability and validity of the measure in identifying a person’s understanding of dementia (Annear, Toye, et al., 2017b).

The Health Literacy Questionnaire (HLQ) was used to examine understanding of health information and navigation of health systems. Nutbeam (Nutbeam, 2000) suggests an approach to operationalise health literacy, with classifications identifying three types of literacy in terms of what literacy enables people to do rather than measures of reading and writing. We used this framework to identify three of the nine subscales to be included in this study so that each literacy classification was reflected. We included; subscale ‘Appraisal of health information’ (HLQ subscale 5/HLQ5) to indicate critical literacy and to capture aspects of health literacy found to be most difficult (Osborne et al., 2013) ‘Navigating the healthcare system’ (HLQ7) to determine communicative/interactive health literacy as communication has been found to influence health knowledge (Andrews et al., 2017), and ‘Understand health information well enough to know what to do’ (HLQ9) to demonstrate basic/functional literacy which was found to be the least difficult of health literacy competences.

Demographic and dementia experience information was collected including carer age, years of education, attendance at a support group (yes/no) and other personal or professional experience of dementia (yes/no). Participant’s postcodes were collected and used to determine deprivation using the Townsend Deprivation Index. Each postcode in the United Kingdom can be categorised as an Index of Multiple Deprivation (IMD) decile from one – most deprived, through to 10 – least deprived). Rurality was also determined from postcodes, with geoconvert (created by the Office of National Statistics) providing classification of; Urban Major Conurbation, Urban City and Town, Urban City and Town in a sparse setting, Rural Town and Fringe, Rural Village, Rural Hamlets and Isolated Dwellings. We combined categories and used Urban Major, Urban City and Town and Rural. The only demographic information of the person living with dementia collected was dementia severity, measured using the proxy rated Clinical Dementia Rating Scale (CDR).

Topic guides were developed as part of the wider project (ref removed for submission (Moore, 2019)) and included questions about grief, preparation for end of life and unmet information needs. For the purpose of this study, data was included which explored what participants perceived to be unmet information and support needs and any programmes or resources that might address these needs (i.e. ‘what is your understanding of how dementia might progress?’ and ‘how have healthcare professionals provided support and information..’).

### Analysis

We thematically and inductively analysed transcripts and field notes to explore how carers’ access information about end-of-life symptoms and emotional support, and the barriers they face in accessing this information. Key themes related to dementia knowledge and carers’ experiences of information resources were identified. Four researchers were involved in the analysis process; * and *removed for review YK and LG assisted with transcribing, *removed for review KM was the experienced qualitative researcher who designed the study and analysis process and oversaw all elements of the analysis, * and *removed for review SC and EF checked the accuracy of transcripts and carried out analysis. Transcripts and summary field notes were uploaded into qualitative software NVivo V11 (QSR International) in order to code chunks of text. Parts of text that seemed important or relevant were labelled with a summary code or phrase that captured the content of text. Relevance constituted carers talking about experiences of talking to Health Care Professionals (HCPs) about dementia, reflections on experience of diagnosis and information needs and changes to need over time, experiences of seeking knowledge and factors that helped or hindered this. Subsequent relevant text was categorised under the same summary code or phrase, and codes that were associated to each other were then grouped together under broader themes. All three researchers initially coded the same transcript and five summary notes and then compared and reviewed codes to ensure consistency in approach. After all transcripts and field notes had been coded, themes were reviewed to reduce duplication and refined further into group concepts.

Participant characteristics were summarised using mean (standard deviation) and proportions as appropriate. Multivariable regression analysis was used to explore the impact of sociodemographic factors on knowledge of dementia using the DKAS (our primary outcome). The regression model contained 12 variables: deprivation (measured by Townsend Deprivation Index); rurality (measured by the rural–urban classification defined by the Office of National Statistics); HLQ7 (navigating the healthcare system); HLQ 5 (appraisal of health information); HLQ9 (understanding health information); carer’s years of education; dementia severity; other experience of dementia (personal and professional); age of carer; relationship with person living with dementia (spouse/partner, adult child or other) and attendance at a carer support group. We aimed to recruit 150 participants to meet the rule of thumb of 10 participants per covariate. We were missing deprivation and rurality data for one participant. Due to the low proportion of missing data for these measures (0.7%), we imputed the missing data using mean imputation.

To identify factors which have the most impact on the total dementia knowledge score we performed a backwards elimination model selection procedure. No consensus exists about the best method for selecting predictor variables, but backwards elimination is generally the preferred method (Heinze & Dunkler, 2017). Factors were eliminated one at a time until variables with *p* < 0.05 remained in the model. Regression analyses were conducted using Stata statistical software, version 15.

## Results

### Participant characteristics

The average age of carers was 63 years (SD = 12.1), and most were caring for a spouse/partner (47%) or a parent (48%) (See Table 2). Carers were well educated with a mean of 16 (SD = 4.0) years of education. Most carers lived in urban areas (89%) and 37% of participants were in the 50% of areas that are most deprived (Table 1). Thirty-two percent cared for someone with severe and 43% for a person with moderate dementia. Four pairs of participants were related to each other (two pairs of siblings and two parent and child dyads), 57% had other personal experience of dementia and 22% had experience of dementia through their occupation.

**Table 1.** Demographic profile.

Categorical variable	N (%)	Mean DKAS (SD.)
All participants	150 (100)	34.8 (7.0)
Recruitment source		
Memory service, mental health service	72 (48.0)	33.3 (6.2)
Join dementia research	58 (38.7)	36.5 (7.1)
Newsletters and other	20 (13.3)	35.4 (8.6)
Gender of carer		
Female	116 (77.3)	34.9 (7.1)
Male	34 (22.7)	34.5 (7.2)
Gender of person living with dementia		
Female	82 (54.7)	34.5 (7.5)
Male	68 (45.3)	35.6 (6.5)
Relationship with person living with dementia		
Spouse	70 (46.7)	33.7 (6.9)
Adult child	72 (48.0)	35.6 (7.1)
Other+	8 (5.3)	38.0 (5.7)
Dementia severity (CDR)		
Mild	38 (25.3)	34.0 (7.6)
Moderate	64 (42.7)	33.8 (7.0)
Severe	48 (32.0)	36.8 (6.3)
Where does person living with dementia live?		
Live at home with participant carer	72 (48.0)	33.4 (7.1)
Lives at home with others/alone	37 (24.7)	35.4 (7.1)
Residential and supported accommodation	41 (27.3)	36.7 (6.4)
Rurality		
Urban major conurbation	70 (47.0)	35.9 (6.7)
Urban city and town	62 (41.6)	34.0 (7.2)
Rural#	17 (11.4)	33.7 (7.6)
Deprivation		
1^st^ (most deprived)	5 (3.3)	32.4 (9.5)
2	9 (6.0)	36.1 (5.9)
3	9 (6.0)	33.3 (2.3)
4	17 (11.3)	36.6 (6.8)
5	15 (10.0)	34.5 (8.1)
6	11 (7.3)	31.9 (7.7)
7	16 (10.7)	35.3 (6.7)
8	17 (11.3)	36.0 (7.3)
9	22 (14.7)	35.1 (6.5)
10^th^ (least deprived)	29 (19.3)	34.5 (7.9)
Other experience of dementia – occupation		
No	117 (78.0)	34.3 (7.2)
Yes	33 (22.0)	36.8 (6.2)
Other experience of dementia – personal		
No	65 (43.3)	34.2 (7.3)
Yes	85 (56.7)	35.3 (6.8)
Attendance at a carer support group		
No	86 (57.3)	35.2 (6.9)
Yes	64 (42.7)	34.3 (7.2)
Knowledge of end-of-life preferences		
No	11 (7.3)	31.2 (7.2)
Yes	110 (73.3)	34.8 (6.9)
Not sure	29 (19.3)	36.5 (7.0)
Formalised documents of end-of-life care		
No	79 (52.7)	34.5 (7.5)
Not sure	8 (5.3)	34.5 (8.1)
Yes	63 (42.0)	35.3 (6.3)
If your relative died soon, how emotionally prepared would you be?^		
Very prepared	41 (28.7)	34.4 (6.6)
Somewhat prepared	56 (39.2)	33.5 (7.1)
Not at all/not sure/declined to answer	46 (32.2)	35.7 (6.9)
If your relative died soon, how practically prepared would you be?^		
Very prepared	82 (57.3)	34.8 (6.9)
Somewhat prepared	38 (26.6)	34.0 (7.2)
Not at all/not sure/declined to answer	23 (16.1)	34.2 (6.7)
Grief		
Low grief (MMCGI- SF <58)	77 (51.3)	35.3 (7.1)
High grief (MMCGI- SF >57)	73 (48.7)	34.2 (6.9)
**Carer variable**	**Mean (SD[range])**	
Age	63.0 (12.1 [28-86])	
Years of education	16.2 (4.0 [8-33])	
HLQ, Appraisal of health information	2.8 (0.6 [1.4-4])	
HLQ, Navigating the health care system	3.4 (0.7 [1.33-5])	
HLQ, understand health care information	4.0 (0.6 [1.8-5])	

+ Included: siblings (*n* = 3), granddaughters (*n* = 2), a niece, a nephew and an ex-spouse; #Includes: Urban City and Town in a sparse setting, Rural Town and Fringe, Rural Village, Rural Hamlets and Isolated Dwellings; ^missing data for seven participants; question added after initial interviews completed. CDR = Clinical Dementia Rating; DKAS = Dementia Knowledge Assessment Scale; MMCGI-SF = Marwit-Meuser Caregiver Grief Inventory Short Form, HLQ = Health Literacy Questionnaire, SD = standard deviation.

Mean DKAS scores were 34.8 (standard deviation (SD) = 7.0, range = 17–48) reflecting eight incorrect or 16 partially incorrect answers out of 25 statements. Whilst almost three quarters of participants correctly identified that dementia is not a normal part of the ageing process, only 39% accurately identified dementia as life shortening (see Table 2 for individual scores).

**Table 2.** Individual items (Dementia Knowledge Assessment Scale).

	False	Probably false	Probably true	True	Don’t know
1. Dementia is a normal part of the ageing process	109 (72.7)	18 (12.0)	9 (6.0)	8 (5.3)	6 (4.0)
2. Alzheimer’s disease is the most common form of dementia	4 (2.7)	11 (7.3)	40 (26.7)	69 (46.0)	26 (17.3)
3. People can recover from the most common forms of dementia	135 (90.0)	14 (9.3)	1 (0.7)	-	-
4. Dementia does **not** result from physical changes in the brain	141 (94.0)	3 (2.0)	2 (1.3)	1 (0.7)	3 (2.0)
5. Planning for end-of-life care is generally **not** necessary following a diagnosis of dementia	113 (75.3)	17 (11.3)	3 (2.0)	10 (6.7)	7 (4.7)
6. Blood vessel disease (vascular dementia) is the most common form of dementia	53 (35.3)	25 (16.7)	21 (14.0)	14 (9.3)	37 (24.7)
7. Most forms of dementia do **not** generally shorten a person’s life	59 (39.3)	44 (29.3)	15 (10.0)	17 (11.3)	15 (10.0)
8. Having high blood pressure increases a person’s risk of developing dementia	23 (15.3)	13 (8.7)	37 (24.7)	41 (27.3)	36 (24.0)
9. Maintaining a healthy lifestyle does **not** reduce the risk of developing the most common forms of dementia	69 (46.0)	38 (25.3)	19 (12.7)	16 (10.7)	8 (5.3)
10. Symptoms of depression can be mistaken for symptoms of dementia	11 (7.3)	10 (6.7)	33 (22.0)	81 (54.0)	15 (10.0)
11. Exercise is generally beneficial for people experiencing dementia	1 (0.7)	-	32 (21.3)	113 (75.3)	4 (2.7)
12. Early diagnosis of dementia does **not** generally improve quality of life for people experiencing the condition	71 (47.3)	32 (21.3)	15 (10.0)	22 (14.7)	10 (6.7)
13. The sudden onset of cognitive problems is characteristic of common forms of dementia	45 (30.0)	26 (17.3)	22 (14.7)	45 (30.0)	12 (8.0)
14. It is impossible to communicate with a person who has advanced dementia	88 (58.7)	29 (19.3)	12 (8.0)	13 (8.7)	8 (5.3)
15. A person experiencing advanced dementia will **not** generally respond to changes in their physical environment	79 (52.7)	23 (15.3)	17 (11.3)	21 (14.0)	10 (6.7)
16. It is important to correct a person with dementia when they are confused	117 (78.0)	14 (9.3)	9 (6.0)	4 (2.7)	6 (4.0)
17. People experiencing advanced dementia often communicate through body language	11 (7.3)	11 (7.3)	33 (22.0)	75 (50.0)	20 (13.3)
18. Uncharacteristic behaviours in a person experiencing dementia are generally a response to unmet needs	25 (16.7)	6 (4.0)	50 (33.3)	44 (29.3)	25 (16.7)
19. Medications are the most effective way of treating behavioural symptoms of dementia. (Missing *n* = 1)	56 (37.3)	28 (18.7)	21 (14.0)	13 (8.7)	31 (20.7)
20. People experiencing dementia do **not** generally have problems making decisions	130 (86.7)	12 (8.0)	3 (2.0)	2 (1.3)	3 (2.0)
21. Movement is generally affected in the later stages of dementia	3 (2.0)	4 (2.7)	30 (20.0)	102 (68.0)	11 (7.3)
22. People with advanced dementia may have difficulty speaking	3 (2.0)	1 (0.7)	8 (5.3)	134 (89.3)	4 (2.7)
23. People experiencing dementia often have difficulty learning new skills	-	4 (2.7)	16 (10.7)	130 (86.7)	-
24. Difficulty eating and drinking generally occurs in the later stages of dementia	2 (1.3)	-	23 (15.3)	119 (79.3)	6 (4.0)
25. Daily care for a person with advanced dementia is effective when it focuses on providing comfort	2 (1.3)	-	32 (21.3)	111 (74.0)	5 (3.3)

Green shading indicates correct answer.

### Regression results

Carer age and relationship to the person living with dementia were highly correlated; therefore, carer age was not included in the multivariable analysis model to avoid issues regarding multi-collinearity.

The initial regression model accounted for just under a quarter of the variation in the outcome (*R*^2^ value = 0.23). Better appraisal of health (coef 3.48, 95% Confidence Interval (CI) (1.38, 5.58); *p* = 0.001) was the only variable associated with higher dementia knowledge scores. There also appears to be an association between understanding health information and dementia knowledge.

In the final model after backwards elimination, in addition to appraisal of health, years of education of the carer was also a significant predictor of dementia knowledge (coef 0.39, 95% CI (0.12, 0.65); *p = 0.004)* (Table 3). 18 percent of the variation in the outcome was explained by the two significant variables (*R*^2^ = 0.18). (Table 4)

**Table 3.** Multivariable regression analysis for total dementia knowledge score (as measured by total DKAS score).

Dementia knowledge – DKAS total score	Coef	95% CI		*p*-value
HLQ subscale: Appraisal of health	3.48	1.38	5.58	0.001
Years of education (carer)	0.25	−0.04	0.54	0.096
Relationship with PwD				
Spouse (ref)	0			0.627
Adult child	0.46	−2.15	3.06	
Other	2.53	−2.64	7.71	
HLQ subscale 7: Navigating the healthcare system	−0.81	−2.77	1.15	0.417
HLQ subscale 9: Understand health information	2.07	−0.29	4.43	0.085
Deprivation	0.22	−0.23	0.67	0.34
Rurality				0.582
Urban major conurbation	0			
Urban city and town	−1.25	−3.78	1.28	
Rural#	−1.51	−5.46	2.43	
Attendance at carer support group				0.624
No (ref)	0			
Yes	0.61	−1.86	3.09	
Occupation working with dementia				0.333
No (ref)	0			
Yes	1.35	−1.39	4.09	
Other personal experience of dementia				0.95
No (ref)	0			
Yes	−0.07	−2.24	2.1	
CDR score				0.146
Mild (ref)	0			
Moderate	0.05	−2.73	2.83	
Severe	2.39	−0.6	5.37	

Coef = coefficient; CI = confidence interval; HLQ = Health Literacy Questionnaire; CDR = Clinical Dementia Rating; ref. = reference group. #Includes: urban city and town in a sparse setting, rural town and fringe, rural village, rural hamlets and isolated dwellings.

**Table 4.** Final model of independent variables most associated with total DKAS score.

	Coef	95% CI		*p*-value
HLQ 5 Appraisal of health	4.06	2.16	5.97	**<0.001**
Years of education (carer)	0.39	0.12	0.65	**0.004**

Coef = coefficient; CI = confidence interval; HLQ = Health Literacy Questionnaire; Using backwards elimination, factors were removed in the following order: 1) Other personal experience of dementia, 2) carer support group, 3) relationship, 4) rurality, 5) deprivation, 6) navigating the healthcare system, 7) other experience of dementia-professional, 8) understanding health information, and 9) Clinical Dementia Rating Score.

### Qualitative findings

Four distinctive themes were identified from the thematic analysis of the interviews and field notes that were related to dementia knowledge. To maintain anonymity of participant data, new participant ID numbers were assigned for the purpose of this paper.

### Arm yourself with information

Carers often described having to seek information for themselves, either in response to information overload at diagnosis or a perceived lack of information provision. Carers sought information using the internet to access organisations, forums, blogs and social media pages and by researching and attending local events and conferences. Taking part in research and reading books on dementia were also avenues carers pursued in order to obtain knowledge.

‘No person has actually taught me anything about dementia in an official way. I have learnt it all from observation from my own research and from people that I’ve encountered in the social care world through campaigning, through charities and social media and other blogs, yeah; through going to conferences, through going with organisations like Dementia UK, Alzheimer’s society, Talking point forum, other bloggers, people I’ve just met informally who work in social care that I chat with, reading up other people’s stories, talking to people’. ID 1, female caring for mother

For some carers, the motivation to seek knowledge was related to feeling prepared and knowing what to expect in the future, for example, one participant (ID2 female caring for mother) described how being ‘forewarned is forearmed’. For others, carrying out research was linked to coping, and the internet and forums were often described as useful in normalising behaviours and experiences and also learning ways of dealing with them.

‘Arm yourself with information from whichever source suits you. Either written information or the internet, so that you do not feel cast adrift’. ID 3, female caring for spouse

The importance of carer ability to steer through information

Carers being able to review and appraise information about dementia were important. Carers described having to dig deep to find information, and that skills were needed to effectively carry out research and interpret information.

‘We were fortunate enough to be able to kind of learn those things and find those things out ourselves by asking the right questions, by looking at the right resources but it didn’t feel that it was as easy to come by’. ID 4, male caring for father

Carers also had to be able to understand information to be able to apply it to their own circumstances, in addition to being able to identify good and reliable sources of information.

‘So there’s information that you can err access and so you know that.. that’s quite helpful but I suppose it does, it’s predicated on you being able to really sort of filter that information, being able to use it, being able to access it, being able to, you know, kind of have the time to do it’ ID 4, male caring for father

‘The main sites used are Alzheimer’s Society, because I just find it to be a trusted source and I would rather go to a single trusted source than start trawling around.…but the problem with the internet is that you can get as much information as you want but you’re never quite sure the validity of that information whereas what I wanted to do was go to the tried and tested site.. where I knew that information would be correct because you know when you go on there you can be given endless reams of information and you’re not quite sure where it has come from or whether it’s even somebody else’s personal thoughts’. ID 5, male caring for brother

### Other experience of dementia can be helpful

Other personal experience of dementia was often perceived as helpful in terms of preparation and knowing that progress is slow. Other experience contributed to being able to identify symptoms of dementia and impacted on understanding even if there were differences to their previous experiences of dementia.

‘Path is almost exactly like her dad so realised it was happening way before anyone else. In some ways, it’s good to know what to expect.. without this experience she would have been quite frightened about what’s coming next, she wouldn’t know that the progress is slow. She can prepare for the next bit as she can see it coming’. ID 6, female caring for spouse. Extract from interviewer notes.

Similarly, experience of working in an occupation relevant to dementia could be perceived as a route into finding relevant information and improving knowledge. Understanding the processes and having the confidence to push and talk to health care professionals and services contributed to being able to navigate the system. However, there was also a sense that working within a dementia related field was very different to caring for a family member or friend.

‘7 years in adult mental health, rest in dementia. Very good insight. Knew what to ask for. But when it happens in your own family it’s so different. Sister was a nurse so that helped too’. ID 7, female caring for mother. Extract from interviewer notes.

### Importance of relationships with healthcare professionals

Carers’ relationships with HCPs, particularly General Practitioners (GPs), also impacted on how and where carers sought knowledge. Some carers spoke of a mistrust in HCPs, having had previous experiences of feeling that their concerns had been dismissed which dissuaded them from seeking further information. Others felt that HCPs lacked knowledge and insight into dementia, and that the system of GP practices (seeing any GP within a practice rather than having one consistent GP) often meant a lack of continuity and relationship building. A small number of carers described being given wrong information by the GP, which created tension and doubt in the GP’s ability to support them with accurate information. Receiving useful information and having face-to-face discussions rather than leaflets also seemed important to carers.

‘They didn't tell us anything; he said ‘I think your dad’s got dementia, mixed dementia. This, this and this and it’s at a moderate stage’; and I thought ‘oh, now what?’, you know and he said ‘well I’m going to give you some information’. Like that much information [a lot]..which ..when you sort it out in the end there’s not very much that is useful.. there was no .. there wasn't enough; I needed more, I needed somebody to sit down and say ‘right’.. with me, just me .. I mean, I know it’s about my dad and I know this .. but you know he wasn't interested in hearing about it but I was .. and I would have questions I would ask that I wouldn't ask in front of my dad’. ID 8, female caring for father

Those who felt they had a good relationship with HCPs tended to feel more supported, with some viewing GPs as the portal of entry for information. Having a single point of contact to answer questions and provide support was also helpful, with one participant (ID 9, female caring for spouse) describing a dementia nurse as ‘my rock’.

‘The people at the memory clinic were very kind. They were… they produced a lot of information for me umm and they were always available if I needed further umm information given so … I would say the back-up was good’. ID 10, female caring for spouse

‘He [person living with dementia] used to have a brilliant GP who really looked after him and losing him was a great loss and [carer] feels they would be better supported if he were still the GP. [Carer] also felt they would be more prepared for the future and have had those discussions if that were the case’. ID 11, female caring for partner. Extract from interviewer notes.

## Discussion

This study provides new insights into the factors that are associated with dementia knowledge among family carers. Integrating findings from quantitative and qualitative data for discussion allows us to provide a more detailed narrative of what influences dementia knowledge among family carers. Under half of the carers interviewed accurately identified dementia as life shortening indicating that notable gaps remain in carer knowledge. This is not surprising considering a recent survey of memory clinics found only 41% of staff routinely describe the nature of dementia as terminal to carers *ref removed for review (Moore, 2019). Almost half of the participants had not formalised end-of-life care documents despite 73% providing care for somebody living with moderate or severe dementia. A lack of awareness and knowledge around the trajectory of dementia could be a contributing factor to documents not being in place.

Of the factors explored, appraisal of health information was most associated with dementia knowledge, with a trend between carers’ ability to understand health information and knowledge also being found. Carers additionally spoke of having to actively seek reliable information; that information was not being routinely provided in a way that was helpful where carers acquire knowledge when seeking information independently is also important to dementia knowledge. We found that attendance at a carer support group was not significantly associated with knowledge, and our findings indicated that those who had attended actually had slightly lower DKAS scores. Therefore, whilst online peer support groups and face-to-face carer support groups may be beneficial in normalising experiences, they may not be reliable or accurate sources of dementia knowledge. This has implications for practice; if there is a lack of trust in health care professionals to provide meaningful information, which was indicated by some participants in this study, this may contribute to carers having to rely on their own independent research. This subsequently relies on the carer’s ability to research effectively and identify reliable sources of information. This reinforces previous work which examined Alzheimer’s disease knowledge across laypeople, patients, carers and professionals; professionals were able to gather information from a range of sources and identify what was most reliable, whilst other groups used fewer resources which were often popular media (e.g. newspaper, television and the internet) or personal contacts, which may not be accurate (Carpenter et al., 2011). Information via the media has been found to focus on diet, physical activity and prevention, and less on symptoms, course and caregiver issues (Mathews et al., 2009). Carers in this study also described misleading or conflicting information, and anecdotal rather than scientific information, highlighting the importance of health literacy. There may be benefit in teaching non-professionals (lay people, patients and carers) skills to firstly locate information and secondly evaluate information about dementia (Carpenter et al., 2011) which is consistent with our findings and other research (Andrews et al., 2017; Stokes et al., 2014).

Years of education received by carers were also found to be a significant predictor of dementia knowledge. This could be related to access to sources of information (Werner, 2001), social class and ability to understand information, as health literacy has been found to play a larger role among those with lower education in terms of health outcomes (Roberts, 2015). Education has also been found to be an important factor in explaining differences in dementia knowledge among ethnic and racial minority groups (ERMG) (Cahill et al., 2015). A consistent finding in the literature is that lower levels of dementia knowledge are common in ERMG (Carpenter et al., 2011); however, we were unable to explore this as only 6% of our sample was from minority backgrounds.

Other experience of dementia, either in a personal or professional capacity, was not found to be associated with knowledge; however, our qualitative findings suggested that carers perceived this as being useful to their knowledge. A possible explanation could be that having other experience may increase confidence, both in interactions with HCPs and navigating the system to access resources, but also in relation to feeling more equipped emotionally with a greater sense of preparedness for the future. Whilst this may not translate into quantifiable knowledge, or the aspects of knowledge measured by the DKAS in the current study, other experience may increase emotional resilience and enable better coping. When reviewing scores across the four domains within the DKAS, the highest mean score was found within care considerations, which measures understanding of dementia symptoms relevant to the provision of care (no statistical test). This reflects findings by Annear et al. 2017 (Annear, Toye, et al., 2017b) who found higher levels of respondent knowledge of care considerations in individuals who were from a nursing background or who identified as family carers relative to other cohorts. This suggests that understanding is delineated by experience and regular interaction with people who live with dementia as well as other factors such as education level (Annear, Toye, et al., 2017b). Furthermore, a study demonstrating the effectiveness of the Understanding Dementia Massive open online course (UDMOOC), suggests that while experiential learning (via education, family or workplace) may contribute to knowledge in some areas, it rarely addresses all of the relevant domains of knowledge (Eccleston et al., 2019).

### Strengths and limitations

The demographic characteristics of our sample are generally representative of the general population providing support to somebody living with dementia; informal carers are likely to be female, a spouse and aged in their sixties (Lewis et al., 2014; Wimo et al., 2013). Although the larger number of females in this sample does restrict our findings for men, 60–70% of people caring for somebody living with dementia in the United Kingdom are female so are finding are generally representable of this population (Alzheimer's Research UK, 2015). Our sample was skewed towards a less deprived and more educated sample, as is common in research studies (Alzheimer’s Disease International, 2014); however, we did have a spread of participants across all 10 deprivation deciles which enabled us to examine the impact of deprivation on knowledge. However, our sample which included 11% of carers living in rural areas may be under representative. Although data for the United Kingdom is limited, there are now greater numbers of older people living in rural areas than urban, with the percentage of older people estimated to be as high as 56% in some rural areas (Alzheimer’s Society, 2018).

Documenting participants’ experiences and comments beyond the structured tick boxes in the first interview provided us with a richer understanding of dementia knowledge for a greater number of people, rather than only relying on the 16 participants who took part in the additional recorded interview. There may be limitations, however, to not directly audio-recording and transcribing all 150 interviews; however limited resources prevented this level of data analysis.

Recruiting carers from clinical teams and JDR may have led to some bias, as carers recruited this way are likely to have had access to support and potentially more information than carers without links to services. One of our qualitative findings also suggests that people interested in research may also be more interested in learning and knowledge, and therefore be more active in seeking out information. Despite this, knowledge of dementia varied with total scores ranging from 17 to 48 out of a possible 50. The wider programme of work in which the data for this study was collected was exploring grief in family carers, and so there may be some bias in clinicians either recruiting carers perceived to be managing well and avoiding carers who might be struggling or vice versa. There was also some difficulty in assessing attendance at a support group as some carers may have only attended once or twice, and previous experience of dementia which could have been a grandparent with whom they had little contact with a long time ago.

A further limitation is that whilst health literacy was identified in the literature as being an important factor to explore, we wanted to include other factors prevalent in the literature such as demographic characteristics and were therefore unable to include all nine subscales of the HLQ.

The DKAS is a validated tool, and whilst there is currently a limited number of previous studies reporting DKAS scores, the scores found in this study are consistent with an international study of 10 countries including the United Kingdom. Whilst this large cohort included the general population and health care professionals, it reports a mean DKAS score of 34.46 from 115 family carers of people living with dementia, so it is culturally interesting to see similar scores across different countries. (Annear, Toye, et al., 2017b; Eccleston et al., 2019). The authors of the DKAS tool have also developed a Japanese version (Annear, Otani et al., 2017a) tested with health students, academics and professionals. Whilst direct comparisons cannot be made due to the adaptations to the tool and differences in sample characteristics, their average score was 57% compared to 70% in our sample, indicating that further research should focus on dementia knowledge in other cultures.

### Future research

The factors explored in this model account for only 23% of variance in dementia knowledge, indicating that other factors contribute to carer knowledge and that there is a continued need to understand the other three quarters of the model. It is not yet known what is considered a good score on the DKAS, but perhaps what is most important is whether the tool captures the knowledge that will be most useful for supporting carers in their roles. Future work could explore this further. Qualitative methods could be used to further understand the role of previous experience of dementia on knowledge that perhaps is not captured by the DKAS. Motivation to seek knowledge emerged from qualitative data and could also be further explored in relation to improving knowledge of dementia. Whether ethnicity influences dementia knowledge was not explored in this study due to sample characteristics, but as there is evidence to suggest lower levels of dementia knowledge are prevalent among ethnic and racial minority groups among the general population (Cahill et al., 2015), this should also be an area of future research. Similarly, gender differences in knowledge and health literacy should be explored. As dementia care is largely provided by females (Annear, Toye, et al., 2017b; Eccleston et al., 2019), male carers may have less experience of interacting with health and social care services. We found DKAS scores to be similar between females and males (no statistical test); however, our sample consisted of only 23% men so further research should explore this further. Health literacy among men is another under researched area. A comparative population study of European health literacy found that men have slightly lower health literacy (Sørensen et al., 2015), but we found minimal differences between male and females on the three HLQ subscales explored, with males scores very slightly higher than females (no statistical test). Findings from a review exploring how internet users find, evaluate and use health information suggest three recommendations to address the issues that can arise from carers carrying out research independently. Professionals should recommend sites, promote more effective search and evaluation techniques and be involved in developing and promoting uniform standards for health information sites (Morahan-Martin, 2004). Such recommendations could be applied to the dementia context.

## Conclusion

Carers are often faced with becoming proxy decision makers as dementia progresses. It is vital that they are equipped with knowledge of the progression and management of dementia in order to ensure appropriate care and maximum quality of life for the person living with dementia in addition to potentially better carer outcomes. We found ability to appraise health information and higher education were associated with better dementia knowledge.

In an information age, vast amounts of information are available, but this in itself brings difficulties. Carers report the need to filter information and discern what is accurate, and associations between health literacy and dementia knowledge have been found. Evidence demonstrates that improved health literacy can build resilience, improve mental health, increase health knowledge and empower people to effectively manage long-term conditions. Community-based peer support approaches have been suggested such as social networks and peer support, to distribute good health literacy which can impact clinical health outcomes and use of services. Other initiatives include health and social care services using the teach-back approach, and empowering professionals through training and inter-disciplinary initiatives. Whilst further research is needed to determine how to best improve health literacy and the cost effectiveness of such initiatives, we recommend that for this population, carer support groups are a potential avenue for provision of more robust information from facilitators who are knowledgeable about dementia.
